# Elevated serum lipid level can serve as early signal for metastasis for Non-Small Cell Lung Cancer patients: A retrospective nested case-control study

**DOI:** 10.7150/jca.48322

**Published:** 2020-10-17

**Authors:** Rixin Li, Bin Liu, Yumei Liu, Yang Liu, Yang He, Duo Wang, Yunxiang Sun, Ying Xu, Qiong Yu

**Affiliations:** 1 Cancer System Biology Center, China-Japan Union Hospital of Jilin University, Changchun, Jilin Province, China.; 2 Department of Epidemiology and Biostatistics, School of Public Health, Jilin University, Changchun, Jilin Province, China.; 3 Department of Hand and Foot Surgery, The First Hospital of Jilin University, Changchun, Jilin Province, China.

**Keywords:** Non-small cell lung cancer, serum lipids, cholesterol, metastasis, nested case-control study

## Abstract

**Objective:** To investigate the association between serum lipid levels in patients with primary non-small cell lung cancer and the risk of developing metastases, a retrospective cohort-based nested case-control study was conducted.

**Material and method:** Patients with primary non-small cell lung cancer admitted to the First and the Third Hospitals of Jilin University from January 2008 through December 2015 were recruited retrospectively based on their electronic medical records. A total of 524 patients were initially considered, consisting of 138 in the case group and 386 as control. Out of these, 110 were finally included in the case group and 110 as control based on additional selection criteria. The following information is collected from all the patients, total cholesterol (TC), low-density lipoprotein (LDL-C), high density lipoprotein (HDL-C) and triglyceride (TG). Logistic regressions were conducted to estimate the odds ratios (ORs) and 95% confidence intervals (95% CI) for non-small cell lung cancer (NSCLC) patients to have metastasis risk when having elevated serum lipid levels. Restricted cubic spline (RCS) curves were used to demonstrate the association between serum lipid levels and the risk of metastasis.

**Results:** Patients with high TC level (*P* = 0.025, 0R = 1.35, 95% CI: 1.03-1.74) and patients with high LDL-C level (Q4: > 3.47 vs Q1: ≤2.54, *P* = 0.002, OR = 3.92, 95% CI: 1.31-11.77) are found to have an increased metastasis risk; and their dose-response relationship was validated by our restricted cubic spline analysis (TC: *P* overall association=0.02, P non-linear association = 0.73; LDL-C: P overall association=0.02, *P* non-linear association = 0.10). These associations were statistically significant, particularly in men who smoked, never drank, and were 65 years of age or younger. In addition, patients with simultaneously high levels of TC and LDL-C have a 60% increased risk of metastasis compared with patients with high levels of TC and normal LDL-C.

**Conclusion:** Dyslipidemia may be a risk factor for metastasis among NSCLC patients. Examination of serum lipid level on a regular basis can provide early signal of metastasis for NSCLC patients.

## Introduction

Lung cancer is the most common type of cancer which is the leading cause of cancer-related death. About 1.8 million people died of lung cancer in 2018, accounting for 18.4% of all cancer-related deaths 1. NSCLC is the main type of lung cancer, accounting for 85% of all lung cancers worldwide 2 which has a poor prognosis and is prone to metastasis. The 5-year survival is about 50% for patients diagnosed with early NSCLC and drops to lower than 5% among patients with metastases 3, and the overall 5-year survival rate for all NSCLC patients is 15% 4. Most patients with advanced NSCLC die within 18 months after diagnosis 4. Therefore, it is crucial to diagnose the disease early, particularly to detect for possible metastasis.

Serum cholesterol levels have been widely used to detect atherosclerosis and cardiovascular diseases. Numerous studies have recently shown that serum lipid levels are associated with tumorigenesis such as lung, breast, and endometrial cancers 5-7. Cholesterol is a key component of mammalian cell membrane used to maintain cell membrane integrity and regulate its fluidity. It has been found that cholesterol plays key roles in cancer cell proliferation and metastasis 8. Low-density lipoprotein (LDL-C) is an important carrier of cholesterol, involved cholesterol transportation. It has been observed that cancer growth depends on exogenous LDL-C, and associates with gene expressions of the LDL-C receptors 9-11. Furthermore, high levels of LDL-C have been found to promote cancer metastasis 12-14.

While an elevated level of total cholesterol (TC) has been found to be a risk factor for cancer metastasis, such as those mentioned above, as well as superficial esophageal and breast cancers 15. No association between elevated (non-cholesterol associated) lipid levels and the risk of cancer metastasis has been widely recognized to the best of our knowledge, except in a few studies which clearly need further investigation 16, 17.

In this retrospective study, we have investigated the association of TC, LDL-C, high density lipoprotein (HDL) and triglycerides (TG) levels with metastasis in primary NSCLC. As far as we know, this is the first study that reports the correlation between serum lipid levels and metastasis of NSCLC. We anticipate that this work will provide assistance to identification of biomarkers that can be used to predict cancer metastasis for NSCLC and possibly other cancers.

## Materials and Methods

### Study design and patients

Based on codes C34 (C34.000, C34.001, C34.100, C34.101, C34.102, C34.200, C34.201, C34.300, C34.301, C34.800, C34.801, C34.802, C34.803, C34.900, C34.901, C34.902) in *the International Classification of Diseases Tenth Revision* (ICD-10), electronic medical records of all patients with primary NSCLC treated at the First and the Third Hospitals of Jilin University between January 1, 2008 and December 31, 2015 were studied. The deadline for follow-up is December 31, 2018, ensuring that each patient is followed for at least 36 months to accurately assess the status of metastasis. The following inclusion criteria are applied:* (1)* patients with primary NSCLC diagnosed by a clinician; *(2)* age 18 or older; and* (3)* the patient was found to have a NSCLC for the first time in one of the two hospitals, without any anticancer treatment. Participants were excluded for the following reasons: *(i)* missing lipid data in their serum chemistry reports; and* (ii)* have other cancers at the same time or a history of cancer.

Patients with lymphatic and distant metastases diagnosed during follow-up for 36 months constituted in the case group, and other NSCLC patients were included in the control group, giving rise to a total of 524 patients, 138 in the case group and 386 in control. All the patients participating in the study were informed at admission that their electronic medical records will be used for scientific research, and signed an informed consent, which was approved by the ethics committee of the school of Public Health, Jilin University.

### Collection of serum lipid indicators and other covariates

The following data were retrieved for each patient: demographic information (gender, age); lifestyle factors (smoking history, drinking history); diagnostic information (cancer TNM, cancer grade); treatment during hospitalization (whether having received radiation or chemotherapy); serum lipid levels at diagnosis (TC, TG, HDL-C, LDL-C); disease history; and family history of cancers. Smoking was defined as smoking at least one cigarette per week for more than 12 months; alcohol consumption defined as drinking at least once a month and continuously drinking for more than 6 months; previous diseases considered include hypertension, diabetes, cardiovascular and cerebrovascular diseases; and the family history of cancer was defined as patients' immediate family having a history of malignant cancers. The patients' lipid data were obtained at the time of the first diagnosis, and were measured using the early morning fasting serum samples.

The following cut-offs are used to define normal vs. abnormal measures: 5.2 mmol / L, 1.7 mmol / L, 1.0 mmol / L, and 3.4 mmol / L for TC, TG, HDL-C, and LDL-C, respectively, according to* the Chinese Adult Dyslipidemia Prevention Guide (2016) 8*. In addition, the lipid data were divided into four quartiles using the following cut-offs: TC (Q1: ≤4.05 mmol/L, Q2: 4.05- 4.57 mmol/L, Q3: 4.57- 5.31 mmol/L, Q4: >5.31 mmol/L); TG (Q1: ≤0.99 mmol/L, Q2:0.99-1.32 mmol/L, Q3: 1.32-1.71 mmol/L, Q4:> 1.71 mmol/L); LDL-C (Q1: ≤ 2.54 mmol/L, Q2: 2.54-2.91 mmol/L, Q3: 2.91-3.47 mmol/L, Q4: >3.47 mmol/L); and HDL-C (Q1: ≤ 0.96 mmol/L, Q2: 0.96- 1.14 mmol/L, Q3: 1.14- 1.38 mmol/L, Q4: >1.38 mmol/L).

### Statistical analysis

When presenting the results of demographic data and clinical indicators, the mean ± standard deviation or median within the range of (25^th^, 75^th^) quartile was used for continuous variables, and the frequency (percentage) was used for categorical variables. Continuous variables between the case and control groups were compared using the Mann-Whitney U test and the T test. The Chi-square tests were used to compare the proportions of categorical variables.

Propensity score matching (PSM) was used to correct for potential confounding factors. We used a 1:1 match between the case and the control groups by using the MatchIt package in R (v3.4.4). The selected matching method was *nearest* with caliper value 0.02. The matching factors were: age, gender, smoking, drinking, whether having received radiation or chemotherapy, previous diseases history, family history of cancers, degree of invasion T, cancer grade, and cancer type. After successful PSM, the conditional logistic regression was used to compare the results of interest between the matching groups. In addition, multivariate logistic regression was used for the unmatched total population. The results of the two groups of data were compared before and after the matching, and a sensitivity analysis was performed to verify the robustness of the results.

Restricted cubic splines (RCS) are known to be able to accurately capture the dose-response relationship between exposure and outcome 18. Hence, we constructed RCS curves for the derived logistic regression model to provide additional validation for the non-linear relationships detected between TC, LDL-C levels and the level of metastasis. The 5^th^, 25^th^, 50^th^, 75^th^, and 95^th^ percentiles of serum lipid levels were used as the predefined knots with reference dosages of TC and LDL-C at 5.2 mmol/L and 3.4 mmol/L. All tests were two-tailed tests, and *P* <0.05 was considered statistically significant. SAS9.4 was used to calculate the RCS curve, and other statistical analyses were conducted using R (v3.4.4).

## Results

### Baseline data

Our PSM analyses of the initially selected 524 NSCLC patient resulted in a matched group of 110 patients in the case group and 110 patients in the control group shown in **Table 1.** In the full population, the average age of the patients was 61.78 (standard deviation: 10.01), and the ratio between the numbers of men and women was 1.79: 1.

We noted significant differences in the degree of cancer invasion (*P* <0.05) and in the radiotherapy or chemotherapy status (*P* <0.05) but no significant differences in age, gender, smoking, drinking, previous diseases history, family history, cancer classification, and cancer type (*P* >0.05) between the case and control groups in the initial group of 524 patients. After PSM, these differences disappeared, and the baseline data between the two groups were highly comparable. It is noteworthy that there were significant differences in the serum levels of TC and LDL-C between the case and the control groups in both the original and the matched populations, with higher levels for patients in the case group.

### Association between serum lipid levels and risk of cancer metastasis

To test the robustness and consistency of our results, we simultaneously did analyses on both the total population and the matched population. We compared the serum lipid levels of patients between the case and the controls as shown in **Table 2.**

After PSM, we note that the risk of metastasis increases significantly with the increase of TC (*P* = 0.025, 0R = 1.35, 95% CI: 1.03-1.74). In the four LDL-C groups, the fourth group (Q4: > 3.47 vs Q1: ≤ 2.54, *P* = 0.02, OR = 3.92, 95% CI: 1.31-11.77) was significantly associated with the risk of metastasis, compared to the first group.

Highly similar results were observed in the initial population of 524 patients. Similarly, high levels of TC (Q4: > 5.31 vs Q1: ≤ 4.05, *P* = 0.009, OR = 3.07, 95% CI: 1.327-7.10), LDL-C (Q4: > 3.47 vs Q1: ≤ 2.54, *P* = 0.023, OR = 2.5, 95% CI: 1.13-5.53) and HDL-C (Q4: > 1.38 vs Q1: ≤ 0.96, *P* = 0.008, OR = 3.82, 95% CI: 1.42-10.27) were significantly associated with metastasis, respectively, in the total population, based on the univariate model, which was corrected for factors such as age, gender, smoking, drinking, status of chemotherapy and radiotherapy, past disease history, family history of cancers, degree of invasion , cancer grade and cancer type.

### Dose-response relationship between TC, LDL-C levels and cancer metastasis risk

**Figure 1:**
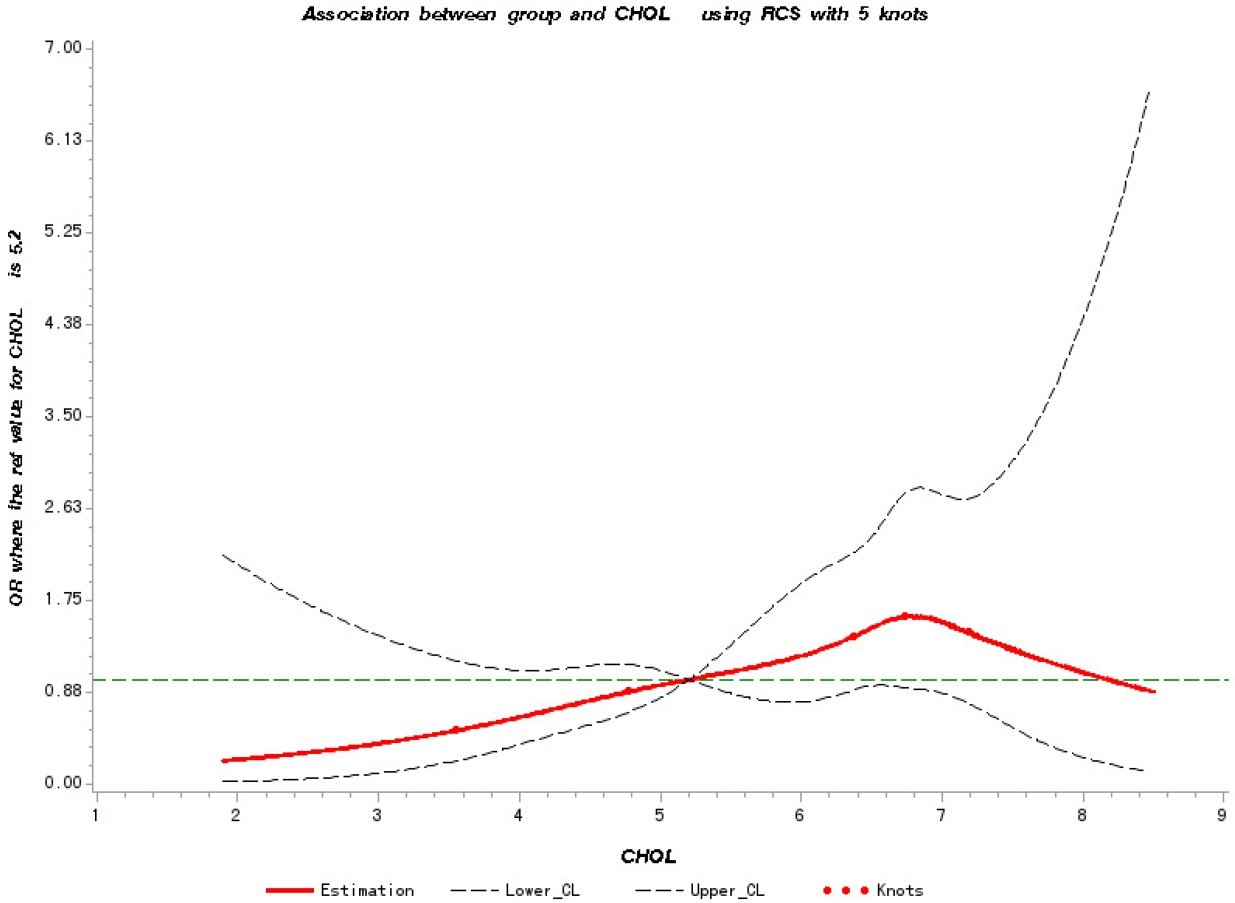
Restricted Cubic Splines for the association of NSCLC metastasis risk with TC. Y-axis indicates the OR of stroke for any value of TC compared with the reference values. Dashed lines refer to 95% confidence intervals.

To further verify the predicted metastatic risk with elevated TC and LDL-C at different levels, we have made RCS charts based on logistic regression. **Figure 1** shows the quantitative relationship between the TC levels in patients and the risk of metastasis. We found that the TC levels are significantly related to the risk of metastasis (test for overall association: *p* = 0.02); and this association is linear (test for non-linear association: *p* = 0.73).We note that the risk of metastasis increases with the increase of the TC level starting at 5.3 mmol/L until the maximum at 6.74 mmol/L, and then decreases from that point on.

**Figure 2:**
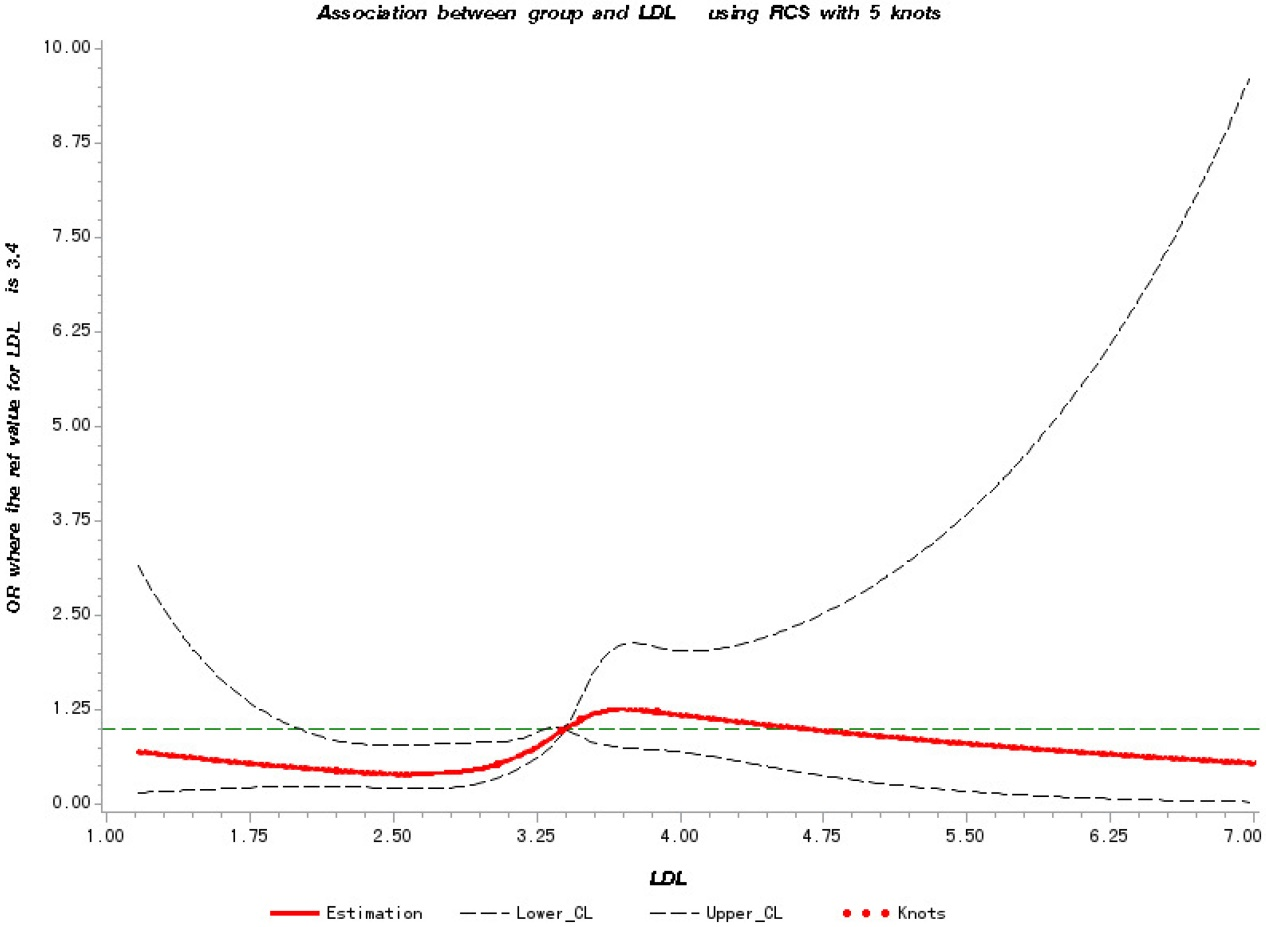
Restricted Cubic Splines for the association of NSCLC metastasis risk with LDL-C. Y-axis indicates the OR of stroke for any value of LDL-C compared with the reference values. Dashed lines refer to 95% confidence intervals.

In **Figure 2**, a significant linear correlation was observed between the LDL-C level and the risk of metastasis (test for overall association: *p* = 0.02; test for non-linear association: *p* = 0.10). And patients have increased risk of cancer metastasis with the increase in their LDL-C level from 3.4 to 4.6 mmol/L.

### Associations between simultaneous elevation in TC and LDL-C and metastasis risk

We have evaluated the risk of metastasis of patients with simultaneous elevation in TC and LDL-C, as shown in **Table 3.** Using normal levels of TC and LDL-C as the reference, patients with normal levels of LDL-C and elevated TC (*p* = 0.013, OR = 2.303, 95% CI: 1.192-4.447) and with simultaneous evaluation of both LDL-C and TC (*p* < 0.001, OR = 2.928, 95% CI: 1.641 - 5.223) were associated with an increased risk of cancer metastasis. Specifically, patients with high LDL-C and high TC have 60% increased risk of metastasis, compared to patients with normal LDL-C and high TC levels.

### Association of TC, LDL-C and metastasis in different subgroup

In a multivariate model with adjusted confounders, we found that there were significant differences in LDL-C, TC levels and cancer metastasis risk between the two genders, but no differences across ages, smoking and drinking statuses, summarized in **Table 4.** Specifically, high TC and high LDL-C were found to be significantly associated with cancer metastasis in men (TC: Q4: > 5.31 vs Q1: ≤ 4.05, OR = 3.81, 95% CI: 1.37-10.64; LDL-C: Q4: > 3.47 vs Q1: ≤ 2.54, OR = 3.27, 95% CI: 1.152-9.289), who smoke (TC: Q4: > 5.31 vs Q1: ≤ 4.05, OR = 4.314, 95% CI: 1.092-17.041; LDL-C: Q4: > 3.47 vs Q1: ≤ 2.54, OR = 5.088, 95% CI: 1.247-20.767), are 65 years or younger (TC: Q4: > 5.31 vs Q1: ≤ 4.05, OR = 6.611, 95% CI: 1.306-33.476, *P* = 0.009; LDL-C: Q4 :> 3.47 vs Q1: ≤ 2.54, OR = 5.182, 95% CI: 1.365-19.676) and the significant association between high TC levels and do not drink (TC: Q4: > 5.31 vs Q1: ≤ 4.05, OR= 3.19, 95% CI: 1.21-8.407).

## Discussion

The present study is the first to suggest a significant association between serum cholesterol level of NSCLC patients and cancer metastasis. Our results revealed that NSCLC patients with simultaneously high levels of TC and LDL-C in the PSM population are at a higher risk of cancer metastasis. And the relationship is linear. The association is particularly significant in men under 65 who smoke but do not drink. In addition, those with simultaneously high TC and LDL-C levels have a higher risk of metastasis than patients having high TC only. These data clearly support the previous studies suggesting that lipids may play a positive role in cancer metastasis.

Our analysis results are supported by the following studies. A retrospective study in 2004 found that high TC is an independent risk factor for lymphatic metastasis in superficial esophageal cancers 19. Among a group of 353 patients with early gastric cancer an increased risk of lymph node metastasis was found to be associated with high TC levels, and this association was more prominent in male patients 9. Similarly, distant metastasis of breast cancer is closely related to hyperlipidemia in patients of breast cancer, and high TC is an independent influencing factor of breast cancer metastasis 10. In addition, a high cholesterol diet promotes cancer growth and metastasis in cancer-bearing mice 11.

Our work suggested that high levels of LDL-C lead to an increased risk of metastasis in NSCLC which is also suggested by a few other studies. A retrospective study of 205 patients with breast, colon, gastric, and ovarian cancers found a significant correlation between high levels of LDL-C (LDL-C> 110 mg/dL) and metastasis (OR = 2.4, 95% CI 1.2-3.5), which further suggested that an increase in LDL level can be used as a predictor for metastatic risk 12. In a study on the prognosis of patients with esophageal squamous cell carcinoma, preoperative LDL-C level plays a crucial role in predicting the prognosis of esophageal squamous cell carcinoma and high levels of such patients, with high level of LDL-C being suggested as promoting lymph node metastasis 13. Furthermore, a study on NSCLC demonstrated that high LDL-C levels at the time of diagnosis were associated with decreased survival of patients 15.

There are several potential mechanisms that may help explaining the association of TC, LDL-C and cancer metastasis. In an oxidative environment like inside cancer cells, cholesterol can be oxidized to oxysterols and further metabolized to a wide range of sex hormones such as estrogen and androgen, all powerful growth factors, hence driving accelerated proliferation of cancer cells. Cancer cells can also combine with activated platelets to form cancer emboli. Studies have shown that LDL-C can induce the expression of ICAM-1, VCAM-1, and P-selectin adhesion molecules in endothelial cells cultured *in vitro*, which can help cancer cells to adhere to serum vessel walls 20. LDL-C is able to affect the immune function of the host as the increase of LDL-C can inhibit the proliferation of T cells, causing immunosuppression and hence potentially leading to the infiltration and metastasis of cancer cells 21.

Factors such as smoking, alcohol consumption, age, and gender have been shown to be associated with tumorigenesis 22-25, but whether any of them may have any impact on cancer metastasis remains elusive. Our study provided strong evidence that high TC and LDL-C levels increase the risk of metastasis in NSCLC. A similar association is revealed between men who are 65 or younger and smoke and never drink. Hence, for such patients, special attention should be paid to the serum levels of TC and LDL-C, which are relatively easy to control via diets and/or medicine, compared to the other factors. Therefore, controlling their serum levels may prove to be an effective strategy for metastasis prevention.

A previous study has detected a linear correlation between the number of dyslipidemia factors (TC, TG, and LDL-C) and increased lung cancer risk 26. This is similar to a result discovered by our study, namely simultaneously increased levels of TC and LDL-C had a 60% increased risk of metastasis compared to patients with increased TC level alone. This provides further guidance for the need to closely monitor the levels of these two factors which may reduce the risk of cancer metastasis NSCLC patients.

In summary, our research here has the following advantages. First of all, we collected the serum lipid samples of the patients from their first diagnosis of NSCLC when they were in the primary stage and have not been treated with any cancer treatment which reduces the effect of drugs on serum lipids. In addition, we collected as much general demographic data, diagnostic information, and treatment status of patients as possible, adjusted all potential confounding factors by the propensity score matching method, and performed a sensitivity analysis in the unmatched total population.

The robustness of our analysis and results are verified. However, there are some limitations in this study. First, because this study is a retrospective study, we excluded all patients who lacked serum lipid tests, which would inevitably cause selection bias. And we found during the information collecting process that some patients' cancer invasion information and cancer grade information are missing, which may cause deviations in the results. Due to the lack of use of lipid-lowering drugs such as statins, some confounding factors may still exist. In addition, the patient's pathological diagnosis was not comprehensive and no stratified analysis was performed in different histological subgroups. Participants are recruited through electronic medical records, while better results will be obtained if outpatients are included. Owing to the lack of financial support, we conducted a retrospective cohort study relying on the electronic medical record system under the existing conditions. In future research, we hope to validate our original findings in an independent prospective cohort. In the end, the impact of individual patient treatment on cancer metastasis had to be considered.

In conclusion, in this retrospective cohort-based nested case-control study, we provided new evidence that high levels of TC and high levels of LDL-C increase the risk of metastasis in NSCLC patients, particularly so for patients with both LDL-C and TC levels elevated. Hence control of LDL-C and TC serum lipids could prove to be a useful strategy for preventing metastasis of NSCLC patients. In addition, controlling serum lipids may also play an important role in slowing down the progression and metastasis of the disease. Further investigation is needed in larger prospective cohort studies to validate our findings.

## Figures and Tables

**Table 1:** Baseline characters in study population

	Full population			Propensity score-matched population		
	Case (N=138)	Control (N=386)	*P* value	Case (N=110)	Control (N=110)	*P* value
Age^a^	61.95 (10.20)	61.72 (9.95)	0.818	61.30 (10.06)	61.46 (9.63)	0.902
**Sex^b^**			0.3			0.902
Male	94 (68.10)	242 (62.70)		69 (62.70)	72 (65.50)	
Female	44 (31.90)	144 (37.30)		41 (37.30)	38 (34.50)	
**Chemotherapy or radiotherapy^b^**			<0.001			0.878
Yes	110 (79.70)	228 (59.10)		82 (74.50)	80 (72.70)	
No	28 (20.30)	158 (40.90)		28 (25.50)	30 (27.30)	
**Smoking status^b^**			0.064			0,787
Yes	65 (47.10)	219 (56.70)		55 (50.00)	58 (52.70)	
No	73 (52.90)	167 (43.30)		55 (50.00)	52 (47.30)	
**Alcohol status^b^**			0.184			1.000
Yes	38 (27.50)	132 (34.20)		34 (30.90)	33 (30.00)	
No	100 (72.50)	254 (65.80)		76 (69.10)	77 (70.00)	
**Previous disease history^b^**			0.688			0.861
Yes	26 (18.80)	65 (16.80)		19 (17.30)	21 (19.10)	
No	112 (81.20)	321 (83.20)		91 (82.70)	89 (80.90)	
**Family history of cancer^b^**			0.152			1.000
Yes	4 (2.90)	3 (0.80)		0 (0.00)	1 (0.90)	
No	134 (97.10)	383 (99.20)		110 (100.00)	109 (99.10)	
T^2^			0.017			0.874
1	13 (32.50)	44 (33.60)		11 (40.70)	13 (44.80)	
2	14 (35.00)	70 (53.40)		14 (51.90)	14 (48.30)	
3	7 (17.50)	5 (3.80)		0 (0.00)	1 (3.40)	
4	6 (15.0)	10 (7.60)		2 (7.4)	1 (3.4)	
NA	98 (71.01)	257 (66.58)		83 (75.45)	81 (73.64)	
**Tumor Grade^b^**			0.457			1.000
1	11 (50.00)	35 (64.80)		10 (76.9)	9 (69.2)	
2	7 (31.80)	11 (20.40)		2 (15.4)	3 (23.1)	
3	4 (18.20)	8 (14.80)		1 (7.7)	1 (7.7)	
NA	116 (84.06)	332 (86.01)		97 (88.18)	97 (88.18)	
**Tumor type^b^**			0.142			1.000
Squamous cell carcinoma	86 (35.80)	268 (46.10)		23 (38.3)	23 (38.3)	
Adenocarcinoma	52 (64.20)	118 (53.90)		37 (61.7)	37 (61.7)	
TC (mmol/L)^c^	6.48 (5.53, 6.81)	6.36 (4.61, 6.74)	0.001	6.48 (5.74, 6.81)	6.37 (4.79, 6.74)	0.041
TG (mmol/L)^c^	1.39 (1.30, 1.61)	1.39 (1.26, 1.50)	0.124	1.40 (1.30, 1.63)	1.40 (1.27, 1.50)	0.328
LDL-C (mmol/L)^c^	3.47 (3.33, 3.52)	3.36 (2.91, 3.48)	0.001	3.47 (3.33, 3.52)	3.36 (3.02, 3.48)	0.022
HDL-C (mmol/L)^c^	1.59 (1.42, 1.65)	1.54 (1.14, 1.65)	0.002	1.59 (1.47, 1.65)	1.55 (1.28, 1.65)	0.069

^a^Mean (Standard Deviation), *p* values from T test; ^b^N (%), *p* values from the X^2^ test; ^c^Median (Interquartile Range), *p* values from Mann-Whitney U test.

**Table 2:** Independent associations of serum lipid levels with risk of NSCLC metastasis

Subgroup	Full population					Propensity score-matched population				
	Case (138)	Control (386)		OR (95%CI)*	*P*_VALUE_	Case (110)	Control (110)		OR (95%CI)	*P*_VALUE_
**TC**					0.002					0.110
Q1	8 (5.80)	49 (12.70)	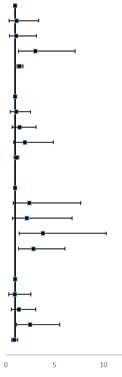	1.00 (ref)		5 (4.50)	12 (10.9)	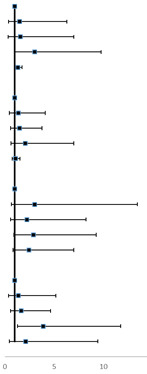	1.00 (ref)	
Q2	9 (6.50)	46 (11.90)		1.13 (0.38-3.38)	0.825	8 (7.30)	12 (10.9)		1.52 (0.37-6.29)	0.560
Q3	12 (8.70)	47 (12.20)		1.12 (0.39-3.17)	0.838	8 (7.30)	13 (11.8)		1.59 (0.36-7.01)	0.540
Q4	109 (79)	244 (63.20)		3.07 (1.32-7.10)	0.009	89 (80.90)	73 (66.4)		3.05 (0.95-9.78)	0.060
continues	6.12 (1.03)	5.72 (1.270)		1.44 (1.17-1.77)	0.001	6.16 (1.01)	5.82 (1.27)		1.35 (1.03-1.74)	0.025
**TG**					0.337					0.690
Q1	12 (8.70)	46 (11.90)		1.00 (ref)		8 (7.30)	12 (10.90)		1.00 (ref)	
Q2	26 (18.80)	88 (22.80)		1.11 (0.48-2.55)	0.808	21 (19.10)	22 (20.00)		1.39 (0.47-4.12)	0.560
Q3	77 (55.80)	209 (54.10)		1.45 (0.67-3.13)	0.344	62 (56.40)	61 (55.50)		1.51 (0.60-3.80)	0.380
Q4	23 (16.70)	43 (11.10)		1.99 (0.81-4.88)	0.133	19 (17.30)	15 (13.60)		2.09 (0.62-7.01)	0.230
continues	1.51 (0.66)	1.46 (0.89)		1.08 (0.86-1.36)	0.507	1.55 (0.71)	1.50 (0.86)		1.09 (0.76-1.56)	0.650
**HDL-C**					0.029					0.330
Q1	5 (3.60)	51 (13.20)		1.00 (ref)		5 (4.50)	12 (10.90)		1.00 (ref)	
Q2	14 (10.10)	45 (11.70)		2.44 (0.78-7.66)	0.125	9 (8.20)	8 (7.30)		3.03 (0.68-13.46)	0.150
Q3	15 (10.90)	45 (11.70)		2.18 (0.70-6.79)	0.179	12 (10.90)	14 (12.70)		2.23 (0.60-8.26)	0.230
Q4	104 (75.40)	245 (63.50)		3.82 (1.42-10.27)	0.008	84 (76.40)	76 (69.10)		2.91 (0.91-9.27)	0.070
continues	1.50 (0.25)	1.41 (0.33)		2.85 (1.34-6.07)	0.007	1.50 (0.25)	1.44 (0.30)		2.45 (0.86-7.03)	0.095
**LDL-C**					0.008					0.040
Q1	10 (7.20)	46 (11.90)		1.00 (ref)		7 (6.40)	14 (12.70)		1.00 (ref)	
Q2	9 (6.50)	47 (12.20)		0.93 (0.33-2.60)	0.885	8 (7.30)	11 (10.00)		1.39 (0.37-5.18)	0.630
Q3	49 (35.50)	160 (41.50)		1.37 (0.61-3.08)	0.440	38 (34.50)	47 (42.70)		1.67 (0.61-4.63)	0.320
Q4	70 (50.70)	133 (34.50)		2.50 (1.13-5.53)	0.023	57 (51.80)	38 (34.50)		3.92 (1.31-11.77)	0.020
continues	2.26 (0.14)	2.43 (2.28)		0.89 (0.63-1.25)	0.493	2.27 (0.14)	2.24 (0.20)		2.11 (0.47-9.46)	0.330

*Univariate Logistic Regression Mode, OR were evaluated by adjusting: age, sex, receiving radiotherapy or chemotherapy, smoking status, alcohol status, previous disease history, family history of cancer, cancer topography, cancer grade, and cancer type.

**Table 3:** Joint associations of TC and LDL-C with NSCLC metastasis risk

Subgroup		OR (95%CI)*
	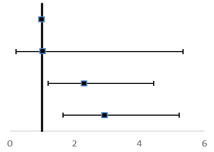	
Normal TC/normal LDL-C		1 (ref)
	
Normal TC/high LDL-C		1.03 (0.20-5.34)
	
High TC/normal LDL-C		2.30 (1.19-4.45)
	
High TC/high LDL-C		2.93 (1.61-5.22)
	

*OR were evaluated by adjusting: age, sex, receiving radiotherapy or chemotherapy, smoking status, alcohol status, previous disease history, family history of cancer, cancer topography, cancer grade, and cancer type.

**Table 4:** The associations of serum lipids with NSCLC metastasis risk by different subgroup status

Subgroup	TC		LDL-C	
	OR (95%CI)*	*P*	OR (95%CI)*	*P*
**Sex**		0.026		0.011
***Male***				
Q1	1		1	
Q2	1.46 (0.39-5.43)		1.32 (0.35-4.98)	
Q3	0.69 (0.17-2.72)		2.28 (0.79-6.59)	
Q4	3.81 (1.37-10.64)		3.27 (1.15-9.29)	
***Female***				
Q1	1		1	
Q2	0.93 (0.10-8.51)		0.53 (0.08-3.42)	
Q3	2.61 (0.39-17.45)		0.69 (0.18-2.69)	
Q4	2.41 (0.46-12.74)		1.40 (0.38-5.18)	
**Age group**		0.666		0.498
***≤65***				
Q1	1		1	
Q2	0.92 (0.11-7.51)		1.64 (0.33-8.13)	
Q3	4.17 (0.69-25.04)		3.56 (0.93-13.59)	
Q4	6.61 (1.31-33.48)		5.18 (1.37-19.68)	
***>65***				
Q1	1		1	
Q2	2.01 (0.39-10.35)		0.55 (0.11-2.78)	
Q3	0.32 (0.06-1.81)		0.69 (0.20-2.35)	
Q4	2.02 (0.65-6.30)		1.55 (0.49-4.92)	
**Smoking status**		0.162		0.118
***Yes***				
Q1	1		1	
Q2	0.90 (0.15-5.46)		1.17 (0.19-7.05)	
Q3	0.30 (0.03-3.60)		2.99 (0.71-12.57)	
Q4	4.31 (1.09-17.04)		5.09 (1.25-20.77)	
***No***				
Q1	1		1	
Q2	1.14 (0.26-5.06)		0.93 (0.24-3.60)	
Q3	1.48 (0.39-5.70)		0.88 (0.30-2.59	
Q4	1.94 (0.62-6.05)		1.45 (0.51-4.09)	
**Alcohol status**		0.261		0.114
***Yes***				
Q1	1		1	
Q2	1.24 (0.11-13.45)		8.42 (0.41-174.58)	
Q3	0 (0-0)		7.54 (0.48-118.09)	
Q4	2.76 (0.43-17.71)		11.84 (0.72-194.42)	
***No***				
Q1	1		1	
Q2	1.22 (0.34-4.35)		0.65 (0.20-2.14)	
Q3	1.41 (0.44-4.52)		1.18 (0.48-2.87)	
Q4	3.19 (1.21-8.41)		2.16 (0.91-5.14)	

*Multivariate Logistic Regression Mode, OR were evaluated by adjusting: age, sex, receiving radiotherapy or chemotherapy, smoking status, alcohol status, previous disease history, family history of cancer, cancer topography, cancer grade, and cancer type.

## Abbreviations

CI: confidence interval
HDL-C: high density lipoprotein
ICD-10: International Classification of Diseases Tenth Revision
LDL-C: low density lipoprotein
NSCLC: non-small cell lung cancer
OR: odds ratio
PSM: propensity score matching
RCS: restricted cubic spline
TC: total cholesterol
TG: triglyceride
